# Morphology-Dependent Optoelectronic Properties of Pentacene Nanoribbon and Nanosheet Crystallite

**DOI:** 10.3390/ma16020557

**Published:** 2023-01-06

**Authors:** Zhifeng Wang, Yuquan Gan, Qianqian Du, Shuhong Li, Yunlong Liu, Wenjun Wang

**Affiliations:** 1 School of Physical Science and Information Technology, Liaocheng University, Liaocheng 252059, China; 2 Shandong Provincial Key Laboratory of Optical Communication Science and Technology, Liaocheng 252059, China

**Keywords:** single crystal, pentancene, morphology

## Abstract

Organic, single crystals have emerged as unique optoelectrical materials due to their highly ordered structure and low defects. In this work, pentacene nanoribbons and nanosheets were selectively fabricated by controlling their growth temperature. The results show that their photoluminescence (PL) activity and electrical properties were strongly dependent on their geometrical morphology and molecular stacking mode such as the degree of π-orbital overlap and intermolecular interaction. The pentacene nanoribbon crystal exhibited a higher PL intensity compared with the nanosheet configuration; conversely, its electrical conductivity was poor. The low-temperature PL measurement indicated that there are stronger π–π stacking interactions in the nanosheet crystal than in the nanoribbon crystal, leading to exciton quenching and higher conductivity. Our study demonstrated that a unique optoelectronic property of organic crystals can be obtained by controlling the crystal’s morphology, which offers potential guidance for the future design and development of organic crystal optoelectronics.

## 1. Introduction

Organic semiconductors have recently attracted intense interest due to their numerous advantages with respect to electronic and photonic devices owing to their easy synthesis and the tenability of their optoelectronic properties brought about by molecular design and engineering [1,2,3,4]. Furthermore, organic semiconductors can be solution-processed on any substrate inexpensively and even at relatively low temperatures, which is highly advantageous for their scale-up from fundamental studies to industrial-level production [5,6,7,8]. Current examples of developed organic semiconductors include field-effect transistors (OFETs) [9,10,11,12], photodetectors (OPDs) [13,14,15,16], solar cells [17,18,19], light-emitting diodes (OLEDs) [20,21,22], and so on. To date, a large number of organic optoelectronic devices have been constructed using amorphous or polycrystalline films [23,24,25]. However, extrinsic structural defects in the films impede their photon, electron, or exciton migration inside the semiconducting layers. Low-dimensional organic single-crystals with a well-ordered structure, low impurity defects, and high carrier mobility provoke greater interest in the field of optoelectronics. Their low-dimensional structure reveals the intrinsic physical properties of an organic semiconductor, thus offering new opportunities to investigate the impacts of basic molecular interactions on structure–property relationships [26,27,28]. Unlike their inorganic counterparts, the optoelectronic characteristics of organic materials strongly rely on molecular (π–π) stacking. In addition, the crystallization rates of different facets govern the macroscopic morphologies of crystals and their products, such as nanosheets, nanodishes, nanorods, and nanowire, and these correspond to the molecular stacking direction. Differently shaped crystals have different applications according to our requirements, even with respect to the same material [29,30,31,32,33,34]. Owing to their desirable optoelectronic properties, high-quality, organic, single-crystalline materials are heavily demanded components in optoelectrical functional devices [13]. Therefore, it is crucial to control the crystal growth process and generate different morphologies, especially along the π–π stacking direction. Over the past few decades, tremendous progress has been made with respect to developing strategies for highly ordered crystal growth, such as vapor and solution-processing techniques [35,36]. Physical vapor transport (PVT) usually produces single crystals with high quality and in dense contact with the substrate. However, this process requires a vacuum or an inert carrier gas environment, which are relatively complicated conditions and incur high costs. For the solution-processing method, it is difficult to fully dissolve organic molecules with a long π-conjugated system in a solvent. Recently, an efficient and simple method with which to evaluate crystalline materials was provided by Ye et al., consisting of micro-spacing in-air sublimation (MAS) growth [37] incurring low costs and yielding crystals with just as good of a performance as other techniques. However, only one shape of organic crystals was studied, and the internal relation of optoelectrical performance with the molecular packing has not been reported. In fact, polycrystalline pentacene has been reported in many references [38,39,40,41]. Previous experiments have developed pentacene with nanoribbon or nanosheet configurations, but the presented analysis of different photoelectric phenomena was insufficiently comprehensive [41,42,43].

In this work, we demonstrated that the shape of a pentacene single crystal can be changed through the growth temperature using the MAS method. The optoelectronic characteristics of pentacene nanoribbons are different from those of the nanosheet configuration. The results show that the PL intensity of the nanoribbon is well above that of the nanosheet, while it shows an opposite phenomenon with respect to electrical conductivity. It is apparent that the optical and electric properties are highly related to a crystal’s shape, which is mainly governed by molecular orientation and packing modes. The different crystals show morphology-dependent properties, which may fulfill different requirements in practical applications.

## 2. Experimental Methods

### 2.1. Materials and Crystals Fabrication

Pentacene powder was purchased from commercial company (Alfa Aesar) without further purification (98% purity). Pentacene crystals were produced using MAS equipment, which has the same setup as the one well-documented in the literature. A silicon wafer substrate was cut into 1 cm × 1 cm pieces, which acted as the substrate for the source holder and for growth of Pentacene crystals. The spacing distance between the two wafers set at 300 μm. For the heating stage, heating temperatures of 180 and 260 °C were employed for the growth of pentacene nanoribbon and nanosheet, respectively.

### 2.2. Characterizations of Pentacene Crystals

Cross-polarized optical microscopy images were obtained by a Zeiss Imager A2m fluorescence-microscope from Carl Zeiss ZESISS, Oberkochen, Germany. Atomic force microscopy (AFM) measurements were performed using a Bruke Icon atomic force microscope from Bruker, Washington, America operating at room temperature and under ambient conditions. X-ray diffraction (XRD) of the crystals was performed with a Panalytical X’pert3 MRD from Malvern Panaco, UK with a Cu Kα anode operating at 40 kV and 40 mA. Unless otherwise stated, all electrical measurements were carried out with a Keithley 4200 from Tektronix, Beaverton, Oregon, America. Parameter Analyzer at room temperature and under ambient conditions. Low-temperature photoluminescence spectra were acquired using a Horiba Jobin Yvon LabRam HR 800 spectrometer from HORIBA Jobin Yvon, Paris, France with a CCD-1024 × 256-FIVS-3S9.

## 3. Results and Discussion

**Figure 1:**
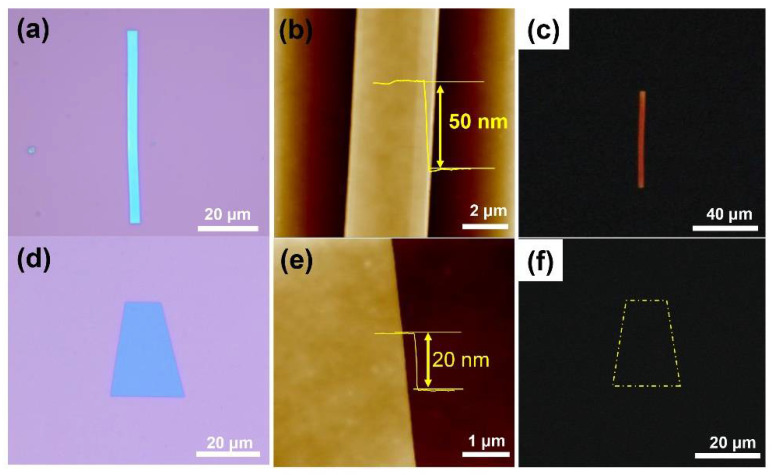
Morphological characterization of the pentacene crystal grown by MAS method. (**a**,**d**) Microscope images of nanoribbon and nanosheet crystals grown at the temperatures of 180 °C and 260 °C, respectively; scale bar: 20 μm. (**b**,**e**) The corresponding AFM images; the inset shows the thickness; scale bars: 2 μm and 1 μm. (**c**,**f**) The corresponding PL images, λ_ex_ = 365 nm, scale bars: 40 μm and 20 μm.

As shown in Figure 1a, the crystals grown at 180 °C display a nanoribbon-like morphology with four right angles. The morphology of the product is highly consistent with previous research [41,42]. The length of the nanoribbon crystal is approximately 60 μm, with a width of ~3 μm (thick ~50 nm, see in Figure 1b). Contrary to fabrication at a lower temperature, a nanosheet with a length and width of 10–20 μm can be obtained at a higher temperature. Its thickness is about 20 nm (Figure 1e). From Figure 1c,f, it can be clearly seen that the nanoribbon has a clear fluorescence signal, while the sheet has no obvious fluorescence intensity. This particular optical difference has previously been reported in a similar way [41,42,43]. The quality of single crystals can be measured by a polarizing test. The obvious brightness variation dependent on the angles offered by the two crystals’ morphologies is shown in Figure S1 (see Supplementary Information), which indicates that the constructed products have high crystallinity.

In order to investigate the growth process of the two different crystals at different temperatures, an experiment consisting of the in situ growth of the crystals under a microscope was conducted. As a traditional silicon wafer is opaque, clean transparent quartz wafers were adopted as the surface-grown crystals. As presented in Video S1, as the temperature of the heating stage increased to 180 °C for one minute, a large number of finely dispersed vapor molecules could be observed on the top substrate. Due to the continuous mass transmission of the bottom substrate, the small crystals continued to grow. This is different from the formation of the nanoribbon crystal. While the other conditions remained unchanged, the growth process of the nanosheet was observed in real time under a microscope, as shown in the Video S2; when the heating temperature reached 170 °C, a small amount of crystal nucleus was formed. However, the growth of nuclei that were inclined to form a morphology corresponding to a nanosheet crystal began, because the growth temperature of a nanosheet crystal is higher than that of nanoribbon crystal. After the heating temperature in the heating stage reached 260 °C, the grains grown rapidly formed nanosheet crystals, while the nanoribbon crystals quickly disappeared due to high intolerance. It can be observed from the Supplementary Videos that the growth rates of the ribbon crystals in the b-axis were significantly higher than those in the a-axis. Unlike the growth behavior of nanoribbon crystals, the growth rates of the nanosheet crystals along the a-axes and b-axes are comparable, resulting in the observed aspect ratio of nanosheet crystals that was lower than that of the nanoribbon crystals.

**Figure 2:**
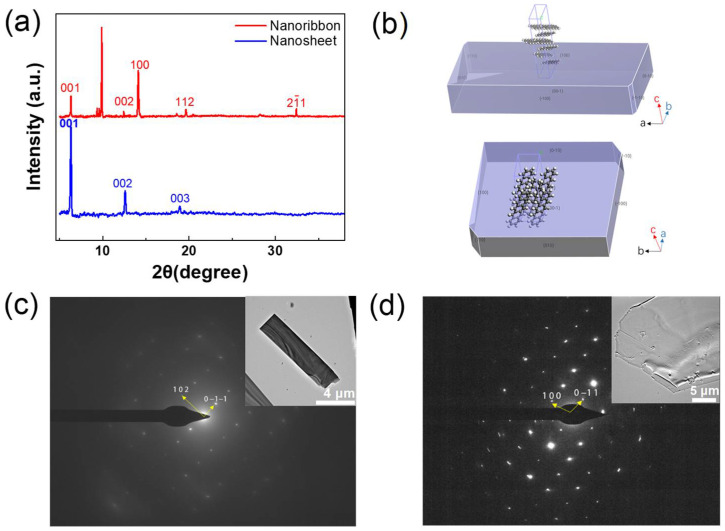
(**a**) XRD patterns of the pentacene nanoribbon crystal (red) and nanosheet crystal (blue). (**b**) Theoretically predicted morphology of the nanoribbon crystal and nanosheet crystal. (**c**,**d**) Selected-area electron diffraction (SAED) pattern images of pentacene nanoribbon and nanosheet depicting zone axes of (21–1) and (011), respectively. The insets are the corresponding TEM images.

Furthermore, the structural characterizations of the as-prepared two types of crystals were carried out using X-ray diffraction (XRD). Figure 2a represents the XRD patterns, which clearly indicates the crystals’ quality and that both the pentacene nanoribbon crystal and the nanosheet crystal are single crystals. The corresponding XRD patterns agree well with the previously reported data concerning pentacene single crystals grown by gas phase transport, as all the patterns show strong diffraction peaks from the plane [41,43]. The theoretical equilibrium morphology of the nanosheets was found in previous theoretical simulations but note that the theoretical morphology of the nanoribbons was lacking [38]. For auxiliary verification, the crystal form prediction of the pentacene material was performed using the BFDH module in the Materials Studio software (as shown in Figure 2b). (The parameters of the crystals have been indexed with lattice constants that were reported previously: a = 0.6266 nm, b = 0.7775 nm, c = 14.530 nm, α = 76.475°, β = 87.682°, and γ = 84.684° [44].) The simulation verified the reliability of the experimental results, which presented crystals with different morphologies.

Meanwhile, the crystals with different morphologies possess different tightness packing modes. The well-defined molecular-packing model and preferential molecular growth behavior in the nanoribbon crystals and nanosheet crystals provide valuable guidance for further investigation of their optoelectronic properties. Individual nanoribbon and nanosheet crystals were characterized by transmission electron microscopy (TEM) (the inset), and their corresponding selected area electron diffraction (SAED) patterns are presented in Figure 2c,d. Typical single-crystal features of nanoribbon and nanosheet single crystal diffraction patterns were obtained. It was clearly shown that the nanoribbon grew along the (0−1−1) and (102). This is different from the pentacene nanoribbon, which showed molecular packing growth along (100) and (0−11).

**Figure 3:**
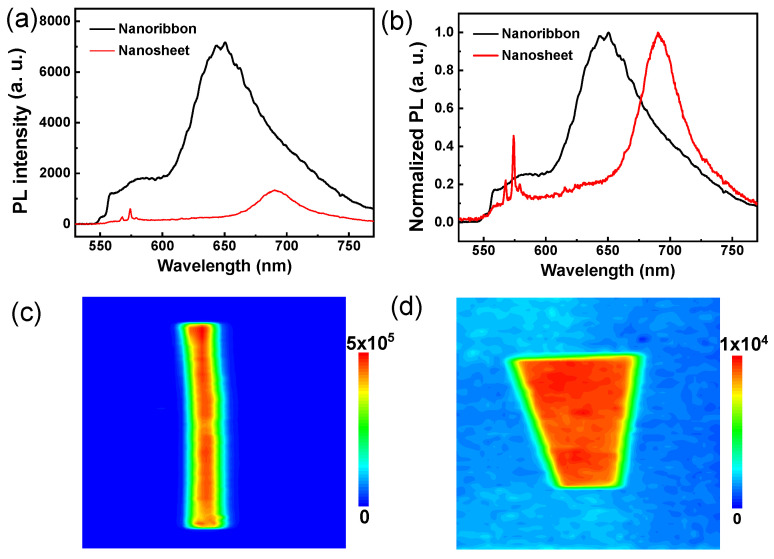
(**a**) PL spectra of pentacene nanoribbon (black) and nanosheet (red). (**b**) The normalized PL data of pentacene nanoribbon (black) and nanosheet (red). (**c**,**d**) PL-mapping images of pentacene nanoribbon crystal and nanosheet crystal, respectively.

As shown in Figure 3a, we found that the nanoribbon showed a stronger PL intensity (λ_ex_ = 532 nm), which was about an order of magnitude higher than that of the nanosheet. This is consistent with the results from the PL images (Figure 1c,f) as mentioned above. The main peak of the nanoribbon is about 650 nm, while that of the nanosheet is about 690 nm, revealing that the energy difference between them is 0.3 eV (shown in Figure 3b). In order to analyze a single crystal that exhibits a strong PL phenomenon, we further investigated the internal arrangement of the crystal. It is worth considering that the existence of pentacene polycrystals has been reported several times in previous reports, and the structure of polycrystals has been explained with respect to different aspects, such as molecular orientation [38,39,40,41,45]. Studies have shown that crystals with different molecular densities show different PL levels [26,27,28,46]. Interestingly, the nanoribbon and nanosheet crystals of pentacene may have distinctly different fluorescence emission properties due to their different molecular-packing models in the solid state. This is because nanosheet crystals exhibit stronger π–π interaction than nanoribbon crystals, resulting in a redshift of their fluorescence spectra. The important factor determining PL activity consists of a crystal’s relative molecular arrangements [47]. Molecular arrangements that favor J-type aggregation are presented in nanoribbon crystals. The difference is that the nanosheet crystals are inclined to H-type aggregation, resulting in a lower PL intensity. To confirm the PL homogeneity of the samples, nanoribbon and nanosheet contour mapping of the PL intensity were performed. The fluorescence intensity distribution of the nanoribbon crystal is consistent with the PL image. Compared with the nanoribbon crystal, the surface fluorescence intensity of the nanosheet crystal is more uniform (as illustrated in Figure 3c,d). The difference in the luminescence intensity indicates that the radiative recombination channel is enhanced by low-temperature crystallization as opposed high-temperature growth [48].

**Figure 4:**
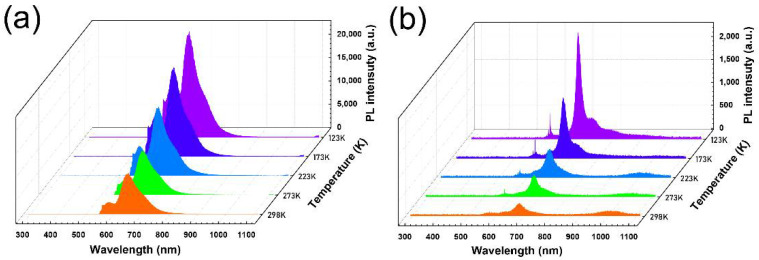
Low-temperature (the range of 123–298 K) PL spectra of (**a**) pentacene nanoribbon and (**b**) nanosheet crystal.

To verify the conjugate intensity of the crystals with different morphologies, a low-temperature (from 123 to 298 K) PL test was carried out. A further exploration for the characteristics of aggregation structures with fluorescence efficiency was carried out. As shown in Figure 4a,b, it is evident that as the temperature decreases, the emission intensity of both crystals increase. This result indicates that the free vibration of molecules inside the crystal could be weakened, and the aggregation state could be reduced at the lower temperature, which increases the intensity of the fluorescence emission. From 298 to 123 K, the changes reveal a 2.6-fold increase in the intensity of the low-temperature spectroscopy in the nanoribbon and a 9.2-fold increase in the nanosheet. This phenomenon suggests that there are stronger molecular interactions inside the sheet crystals at room temperature, while the interactions in the ribbon crystals are weaker due to the larger intermolecular distance [47,49,50,51]. The luminescence properties of organic solid-state materials depend heavily on intermolecular interactions, and small changes in the intermolecular aggregation structures can produce large changes in luminescence properties [52,53,54]. Intermolecular interactions play an essential role with respect to the PL properties of organic, solid state, luminescent materials. Shorter molecular packing leads to tighter intermolecular interactions, contributing to the weaker emission in the longer wavelength region. We concluded that a tighter packing structure causes red-shifting in the spectrum of the nanosheet crystal, which is consistent with the cause of the fluorescence emission. Our findings will lay a foundation for the development of high-performance organic luminous materials.

**Figure 5:**
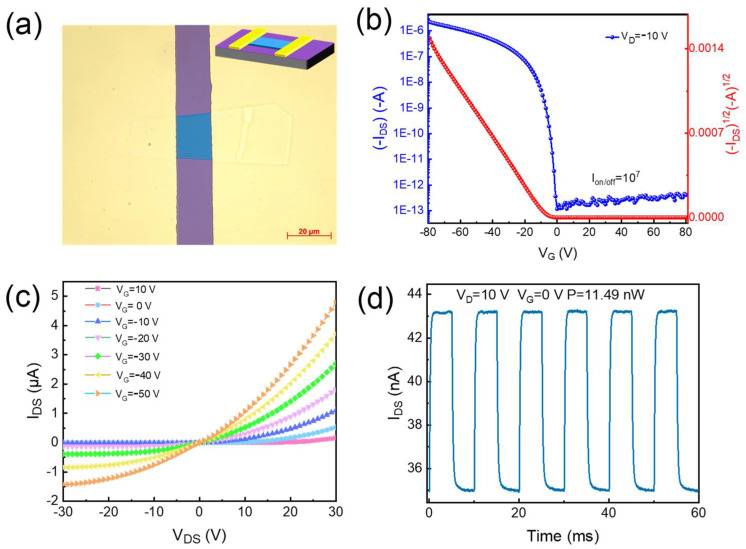
(**a**) Optical image of nanosheet crystal based on OFETs. (L = 15 μm, W = 19 μm.) Inset shows a schematic of the device’s configuration. The representative (**b**) transfer curves and (**c**) output curves of OFET based on nanosheet crystal. (**d**) Time-dependent photoresponse of the device under 658 nm illumination (V_DS_ = 10 V, V_G_ = 0 V).

To investigate the charge transport properties of the grown pentacene single crystals, field effect transistors were prepared based on the pentacene nanoribbon and nanosheet’s micro-crystalline structures. As shown in the inset of Figure 5a, a top-contact bottom gate architecture was adopted. To ensure a clean conductive interface, two Au electrodes were mechanically transferred on both sides of the pentacene nanosheet as source/drain contact electrodes. The representative optical micrograph of the OFET based on the nanosheet single crystal is shown in Figure 5a. At the same time, we fabricated OFET based on nanoribbon crystals; no field effect properties were detected (see Figure S2 in the Supplementary Materials). The above-mentioned different electrical phenomena of the different crystals can be ascribed to two aspects: (1) poor contact property with the electrode due to defects on the crystal surface and (2) the weak π–π interactions in the crystal. Another reason is the poor quality of the single crystals grown at low temperature, which can be verified from the wide PL spectrum. To further investigate why nanoribbon crystals do not conduct electricity, their KPFM parameters were tested. The potential diagrams indicate that the potential of the nanosheet crystal is higher than that of gold; however, the potential in the region of the nanoribbon crystal is lower than that of Au. It can be concluded that the uneven potential inside the nanoribbon crystal causes the phenomenon in which a steady current is not formed (see Figure S3 in the Supplementary Materials). Typical p-type field effect modulation was attributed to the nanosheet single crystals as evidenced by their representative transfer curves attained on the OFET device (Figure 5b). It can be surmised that there is good contact between the electrode and the pentacene nanoribbon crystal (Figure 5c), which is due to the good degree of matching between the Fermi level of the gold electrode and the pentacene single crystal. In contrast, the field-effect performance with respect to the OFET based on the nanoribbon crystal was not measured in this experiment. It can be seen from Figure 5c that the source-drain (I_DS_) increases with the negative increase in the source-drain voltage (V_DS_) and the gate voltage (V_G_). At the same time, it can be seen from the transfer characteristic curve of the device that at V_DS_ = 10 V, the device’s “on/off” current ratio (I_on/off_) is 10^7^, which exhibits a typical p-type field effect modulation. The transistor exhibits an excellent photocurrent upon its illumination with excitation light (658 nm) under a constant bias voltage (V_DS_ = 10 V; V_G_ = 0 V; shown in Figure 5d). The carrier mobility histograms corresponding to the as-prepared 10 devices are illustrated in Figure S4 in the Supplementary Information, showing an average mobility of 0.46 cm^2^ V^−1^ s^−1^ and the highest mobility of 1.2 cm^2^ V^−1^ s^−1^.

## 4. Conclusions

In this work, pentacene nanoribbon and nanosheet crystals that exhibit completely different optical and electrical properties were selectively prepared using the microspace sublimation method. This was facilitated by the different molecular arrangement characteristics provided by the different crystals. At the same time, we verified the existence of different forms of pentacene by theoretical simulation experiments. Furthermore, a low-temperature PL test was carried out to verify the phenomenon in which molecular packing governs intermolecular interactions. It is worth noting that field effect transistors are prepared based on nanoribbon crystals and nanosheet crystals. Unlike a strip crystal device, a device based on a sheet crystal offers good charge transfer characteristics. Moreover, such a device exhibits an excellent photocurrent upon its illumination. The discovery of this particular photovoltaic phenomenon has important implications for future applications, such as the development of organic semiconductor-based optical waveguides and photodetectors.

## Data Availability

The data presented in this study are available on request from the corresponding author.

## Supplementary Materials

The following supporting information can be downloaded at: https://www.mdpi.com/article/10.3390/ma16020557/s1, Figure S1: The cross-polarized optical microscopy images of the nanoribbon crystal and nanosheet crystal in different polarization directions; Figure S2: Microscopy image of a representative nanoribbon crystal device, and the corresponding transfer curve of device based on the nanoribbon crystal; Figure S3: The KPFM images of the nanoribbon crystal device and the nanosheet crystal device and the corresponding surface potential profiles.; Figure S4: Carrier mobility distribution of 10 OFETs fabricated on pentacene sheet-like single crystals. The Video S1: The growth process of the nanoribbon crystals. The Video S2: The growth process of the nanosheet crystals.
