# Supplementary material for: Morphology-Dependent Optoelectronic Properties of Pentacene Nanoribbon and Nanosheet Crystallite
Source: Materials (Basel). 2023 Jan 6;16(2):557. doi: 10.3390/ma16020557 (PMC9864833; doi:10.3390/ma16020557)
Supplement: Supplementary file 1 [file materials-16-00557-s001.zip › materials-2126288-SI.pdf]

Supporting material

Morphology dependent optoelectronic properties of pentacene nanoribbon and nanosheet crystallite

Zhifeng Wang[1, 2], Yuquan Gan[1, 2], Qianqian Du[1, 2*], Shuhong Li[1, 2], Yunlong Liu[1, 2] and Wenjun Wang [1, 2*]

1 School of Physical Science and Information Technology, Liaocheng University, Liaocheng 252059, China
2 Shandong Provincial Key Laboratory of Optical Communication Science and Technology, Liaocheng 252059, China
* Correspondence: duqianqian@lcu.edu.cn (Q.D.); wjwang@lcu.edu.cn (W.W.)

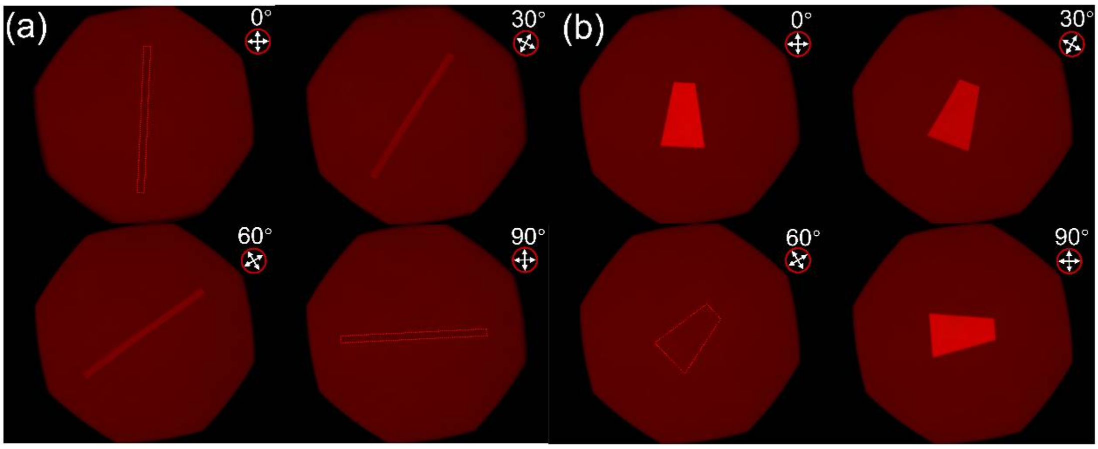

Figure S1. The Cross-polarized optical microscopy images of (a) the nanoribbon crystal and (b) nanosheet crystal in different polarization directions.

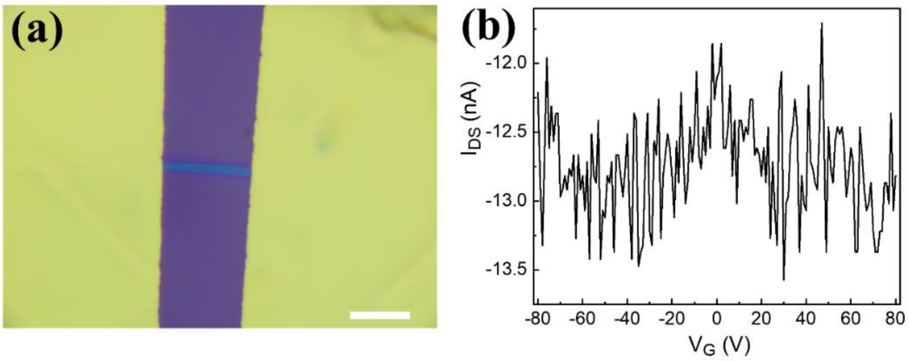

Figure S2. (a) Microscopy image of a representative nanoribbon crystal device. Scale bar: 5 μm. (b)The corresponding transfer curve of device based on the nanoribbon crystal.

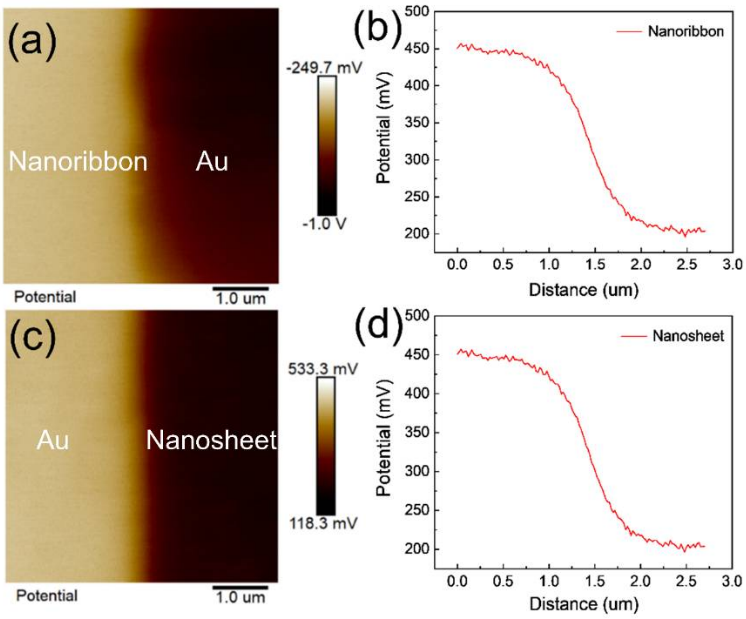

Figure S3. The KPFM images of (a) the nanoribbon crystal device and (c) the nanosheet crystal device, respectively. The corresponding surface potential profiles of (b) the nanoribbon crystal device and (d) the nanosheet crystal device.

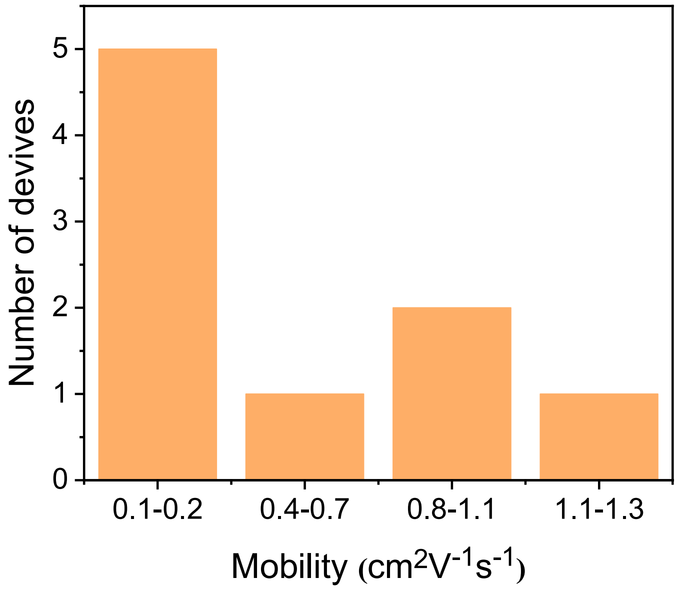

Figure S4. Carrier mobility distribution of 10 OFETs fabricated on pentacene sheet-like single crystals.
